# Evaluation of Five Methods for Total DNA Extraction from Western Corn Rootworm Beetles

**DOI:** 10.1371/journal.pone.0011963

**Published:** 2010-08-13

**Authors:** Hong Chen, Murugesan Rangasamy, Sek Yee Tan, Haichuan Wang, Blair D. Siegfried

**Affiliations:** Department of Entomology, University of Nebraska-Lincoln, Lincoln, Nebraska, United States of America; Institute of Evolutionary Biology (CSIC-UPF), Spain

## Abstract

**Background:**

DNA extraction is a routine step in many insect molecular studies. A variety of methods have been used to isolate DNA molecules from insects, and many commercial kits are available. Extraction methods need to be evaluated for their efficiency, cost, and side effects such as DNA degradation during extraction.

**Methodology/Principal Findings:**

From individual western corn rootworm beetles, *Diabrotica virgifera virgifera*, DNA extractions by the SDS method, CTAB method, DNAzol® reagent, Puregene® solutions and DNeasy® column were compared in terms of DNA quantity and quality, cost of materials, and time consumed. Although all five methods resulted in acceptable DNA concentrations and absorbance ratios, the SDS and CTAB methods resulted in higher DNA yield (ng DNA vs. mg tissue) at much lower cost and less degradation as revealed on agarose gels. The DNeasy® kit was most time-efficient but was the costliest among the methods tested. The effects of ethanol volume, temperature and incubation time on precipitation of DNA were also investigated. The DNA samples obtained by the five methods were tested in PCR for six microsatellites located in various positions of the beetle's genome, and all samples showed successful amplifications.

**Conclusion/Significance:**

These evaluations provide a guide for choosing methods of DNA extraction from western corn rootworm beetles based on expected DNA yield and quality, extraction time, cost, and waste control. The extraction conditions for this mid-size insect were optimized. The DNA extracted by the five methods was suitable for further molecular applications such as PCR and sequencing by synthesis.

## Introduction

DNA extraction is a routine step in many biological studies including molecular identification, phylogenetic inference, genetics, and genomics. In addition, DNA extraction is often used in medical examinations, clinical diagnostics, and forensic investigations. Therefore, a variety of methods have been established to isolate DNA molecules from biological materials [1], and many DNA extraction kits are commercially available.

Different methods have various effects on DNA extraction [2], [3]. An ideal extraction technique should optimize DNA yield, minimize DNA degradation, and be efficient in terms of cost, time, labor, and supplies. It must also be suitable for extracting multiple samples and generate minimal hazardous waste.

The sodium dodecyl sulfate (SDS) and cetyltrimethyl ammonium bromide (CTAB) methods are commonly used for DNA extraction from diverse organisms [1]. These two methods are relatively time-consuming and require a fume hood to operate because of the phenol and chloroform involved. DNAzol® involves a single extraction buffer that solubilizes all cellular components and allows selective precipitation of DNA in the presence of ethanol [4], [5]. The Puregene® Kit contains two solutions to isolate DNA [6]. DNA is first released by lysing the cells with an anionic detergent in the presence of a DNA stabilizer. Proteins and other contaminants are removed by salt precipitation. Finally, DNA is precipitated with ethanol. The DNeasy® Mini Procedure uses a spin-column of DNA-binding membrane and a buffer system for cell lysis, DNA binding and elution [7].

With the presence of sodium ions, absolute ethanol or isopropanol are commonly used to precipitate DNA from its aqueous solution. There are tremendous variations in volume of ethanol or isopropanol (1–2.5x volume of supernatant with DNA), incubation temperature (−80–25°C) and time (0–15 hr) used for DNA precipitation [2], [3], [8]–[18]. However, longer incubation time and lower temperature did not enhance precipitation of Herring sperm DNA sonicated to a range of 200–400 bp when its concentration was ≥5 ng/µl [19], [20].

Western corn rootworm beetles, *Diabrotica virgifera virgifera*, is important because of its status both as an important pest of maize cultivation and as an invasive species in North America and Europe. Thus, DNA extraction is involved in a variety of applications related to the beetle's genetics, genomics, parasite detection, molecular toxicology, and molecular mechanisms of resistances to insecticides and transgenic *Bt* corn [21]. We have noticed that the quantity and quality of DNA isolated from individual rootworm beetles varied considerably among the various extraction methods, and therefore, a comparison of the various methods was conducted to optimize DNA extraction. In this study, we evaluated the DNA yield and quality using the five methods and compared the cost and time required for processing each sample among the methods. To assess the quality of DNA from different methods for PCR application, amplifications of six microsatellite loci at various positions in the beetle's genome were tested for each method. We also investigated the effects of ethanol volume, temperature, and incubation time on DNA precipitation. The factors affecting DNA yield and quality were discussed. Our goal is to evaluate these DNA extraction techniques for handling of a large number of small animals such as insects.

## Results

### Body weight and DNA yield

Although the ranges of body weight overlapped between the females (8.0–19.8 mg) and males (6.1–11.6 mg), females (mean ± SE, 12.22±0.17) were significantly heavier than male's (8.23±0.27) (T  = 5.24, df  = 30 P<0.001). Significant linear regressions between body weight (x mg) and DNA yield (y ng) existed only for the SDS and CTAB methods (SDS: y  = 4716x–16836, R^2^  = 72.4%, P<0.01; CTAB: y  = 3187x–9187, R^2^  = 66.8%, P<0.01).

### DNA yield rate and absorbance ratio

Yield rates and absorbance ratios for the five methods of DNA extraction are listed in Table 1. The extraction method had a significant effect (F  = 12.62, df  = 4, P<0.01) while the gender as a factor was not significant on the yield rate (F  = 0.23, df  = 1, P>0.05). The yield rates by the SDS and CTAB methods were significantly higher than those obtained by the DNAzol® Reagent, Puregene® Kit, and DNeasy® Kit (Tukey's, P<0.05).

**Table 1:** DNA yield rate, absorbance ratio, DNA pellet color, and estimated cost and time used for one beetle extraction by five extraction methods.

	SDS	CTAB	DNAzol®	Puregene®	DNeasy®
DNA yield rate (mean ± SE) (ng/mg)	2982±318	2200±246	1568±126	1328±153	1229±88
Absorbance ratio (mean ± SE) and range	1.98±0.021.91–2.05	2.01±0.041.72–2.10	2.09±0.012.04–2.17	1.89±0.041.72–2.07	1.96±0.021.84–2.05
Color of DNA pellet	light to dark brown	clear to white	light to dark brown	yellow to brown	clear to white
Estimated cost (USD) and time (hr) per sample	0.62–0.863.3	0.63–0.873.3	1.43–1.623.3	0.84–0.912.8	2.68–2.721.3

The means absorbance ratios for all the five methods were higher than 1.8. The mean ratio mean of Puregene® was most closest to 1.8 while the DNAzol® ratio mean was the highest, indicating the lowest and highest protein contamination among the five methods, respectively. Although gender did not have a significant effect on the absorbance ratio, it was significantly affected by the five extraction methods (F  = 7.65, df  = 4, P<0.01). Statistically, the ratio mean of DNAzol® was higher than those of the SDS method, Puregene® Kit, and DNeasy® Kit, and the ratio mean of CTAB method was higher than that of DNeasy® Kit (Tukey's, P<0.05).

### Colors of DNA pellet

The color of precipitated DNA pellets were different within each DNA extraction method but varied more widely across the methods (Table 1). The pellet colors, ranging from clear, white, yellow to brown, did not correspond to the absorbance ratios of DNA in the individual tubes. Therefore, the color of DNA pellets did not indicate the levels of protein contamination in this study.

### Electrophoresis analysis of extracted DNA

**Figure 1:**
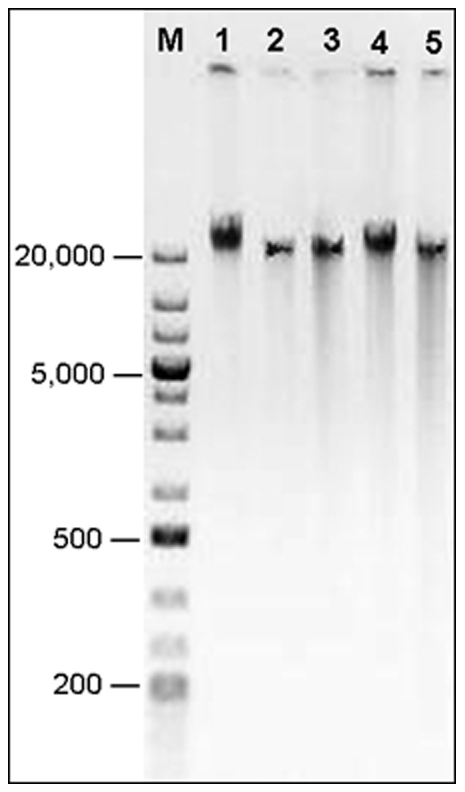
DNA electrophoresis on 0.5% agarose gel at 45 volts for 2 hrs. GeneRuler™1 kb Plus DNA markers (M, in bp) and typical DNA samples isolated by SDS (1), CTAB (2), DNAzol® (3), Puregene® (4) and DNeasy® (5).

Typical examples of DNA extraction using the five methods visualized on a 0.5% agarose gel are presented in Fig. 1. The main bands of DNA were around 40 kb in size. Compared to the three commercial kits, the SDS and CTAB methods showed relatively lighter smear tails, indicating less DNA degradation.

### PCR amplification of microsatellite loci

The core set of six microsatellites, ranging between 182–249 bp, were all successfully amplified from the 10 DNA samples extracted by each method (data not shown), indicating that the five methods isolated DNA of sufficient quality for PCR application.

### Cost and time consumed

The estimated cost in US dollar (USD) and time in hours for each method to extract DNA from a single beetle are listed in Table 1. Due to the much lower expenses of the laboratory-prepared SDS and CTAB buffers (approximately, 0.01–0.02 USD per sample), these two methods were less costly than the three commercial kits. The DNeasy® kit was the most expensive among the five methods but required the least amount of extraction time.

### DNA precipitation

Although the incubation periods had little effect on DNA precipitation, the ethanol volumes and temperatures both had a significantly-positive effect on DNA yield rate obtained by precipitation (ethanol: F  = 21.51, df  = 2, P<0.01; temperature: F  = 20.71, df  = 2, P<0.01). Significant differences existed among the DNA yield rates resulting from various ethanol volumes (60 vs. 120 vs. 240 µl) or temperatures (4 vs. −20 vs. −80°C) used for the precipitation (P<0.05). Among the precipitation conditions tested in this study, 240 µl of chilled ethanol (8x volumes of the SDS supernatant) and centrifugation immediately after ethanol addition, resulted in the highest DNA yield rate.

**Figure 2:**
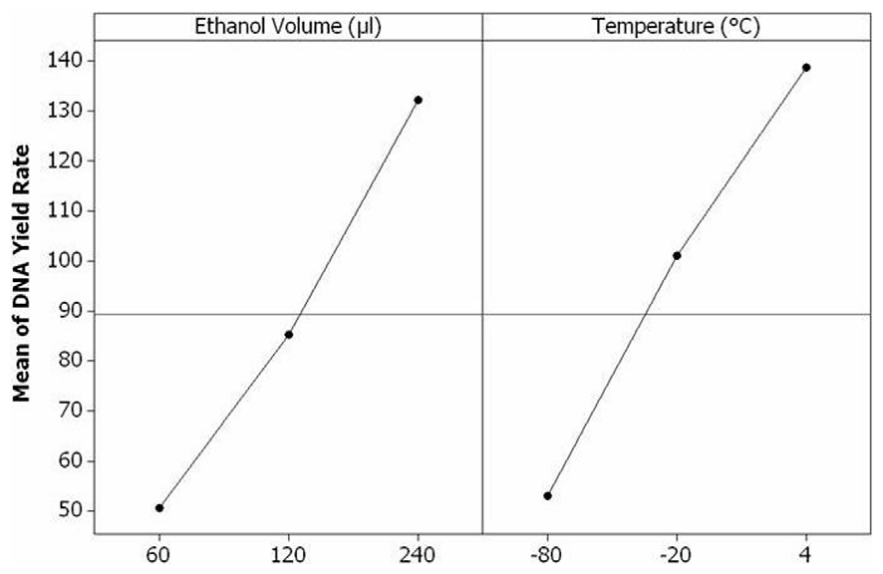
Main effects plot of ethanol volume and temperature on DNA yield rate resulted from precipitation. The horizontal reference line is drawn at the overall mean of DNA yield rate. The dots display the response means for each factor level. The effects are the differences between the means and the reference line.

The main effects plot (Fig. 2) showed magnitudes of the various levels of ethanol volume and temperature for DNA precipitation, indicating the optimal conditions of 240 µl ethanol and 4°C in this study.

## Discussion

DNA yield rate, protein contamination, and DNA degradation in the individually-extracted DNA samples from individual beetles varied within each extraction method and across the five methods, although all the methods isolated considerable amounts of DNA with acceptable quality. Compared to the three commercial kits, the SDS and CTAB methods using laboratory-prepared buffers, resulted in higher DNA yield rates and less degradation. The DNA extracted by the Puregene® Kit contained the lowest protein contamination while the DNA isolated by the DNAzol® and CTAB methods had relatively higher protein concentrations as indicated by the absorbance ratio. With the shortest time spent for a single extraction, the DNeasy® Kit was the most convenient. In general, the three commercial kits did not generate hazardous waste containing phenol and chloroform and did not require a fume hood to operate.

For animal tissue, typical yield rates ranged from 1000–5000 ng/mg [17]. DNA yield is influenced by many factors such as species, tissue, method of preservation, extraction procedure, and precipitation method. The yield rate for abdomens of the tobacco budworm, *Heliothis virescens*, by the CTAB method, was between 500–600 ng/mg [12]. From integument tissue of the sea buckthorn carpenter moth, *Holcocerrus hippophaecolus*, 2000–3000 ng/mg was obtained using a modified SDS method [3]. For the western corn rootworm beetles in this study, all five extraction methods generated acceptable yield rates, ranging from 710 ng/mg by the DNeasy® to 4466 ng/mg by the SDS method. However, only the SDS and CTAB methods showed significant linear regressions of body weight (mg) on DNA yield (ng). The limited extraction rates and higher degradation of the other three methods might have distorted such relationship.

The quality and quantity of extracted DNA could be affected by the incubation temperature for lysates. Using the CTAB method, Shahjahan et al. [12] found that the incubation at 37°C resulted in more than double the amount of total DNA and the lowest mean absorbance ratio (1.72), compared to the incubations at 19, 65 and 80°C, although the isolated DNA was in acceptable quantities and qualities regardless of the temperature. Higher temperature for lysis could also cause DNA degradation [22]. However, the effect of lysis incubation at 37°C needs to be verified for other organisms because higher temperature ranging 55 to 65°C are commonly used in the SDS and CTAB methods [1], [3], [9], [15], [18].

The colors of DNA pellets obtained at the end of extractions did not indicate the levels of protein contamination in this study. The colors varied among extraction methods using different reagents and within each method, probably due to the status of biological materials used for DNA extraction.

Regarding the DNA quality for molecular application, all five methods can provide sufficient DNA for PCR as demonstrated by the microsatellite amplifications in this study. With an estimated size of 2.5 Gb, the beetle's genome has been proposed to be sequenced using the novel parallel sequencing technologies including sequencing by synthesis (SBS) such as Illumina®, SOLiD™ and 454 [23]. To prepare the DNA library, extracted genomic DNA needs to be fractioned into smaller fragments (27–42 bp for Illumina®, 50–75 bp for SOLiD™, and 300–500 bp for 454) [24]–[26]. Therefore, the low levels of DNA degradation during the extractions (most fragments >300 bp, Fig. 1) should not affect the DNA application in the SBS. For molecular detection of *Wolbachia*, bacteria infection in individual beetles using 4 sets of primers [27], the DNA samples with concentrations lower than 100 ng/µl tended to give more false negatives in PCR amplification (1–2 µl DNA template in 10 µl volume of PCR reaction; HC, unpublished data). Therefore, we recommend the SDS and CTAB extractions for *Wolbachia* detection. In general, an extraction method should be tested for the follow-up molecular application before a large-scale extraction of DNA.

To estimate the cost of DNA extraction for certain number of samples via different methods, one can use the cost for each method in Table 1 multiplied by the number of samples to be extracted. However, the time estimated in Table 1 must be adjusted by the times of incubation and centrifugation used to finish all of your samples. In this study, we did not count the time spent for buffer preparation in the SDS and CTAB methods, but this time should be considered when extracting DNA from a few samples.

The higher volume of ethanol (up to 8x volume of the aqueous supernatant containing DNA) and temperature (4°C) in this study enhanced the yield of DNA precipitation. Higher volumes of ethanol facilitate precipitation when the expected DNA concentration is high but may not be suitable for DNA precipitation at lower concentrations because the larger volume slows down the movement of the DNA aggregation during centrifugation [19], [20]. Using a larger volume of ethanol also increases the extraction cost. Generally, 2–3x volume of ethanol is recommended [1], [3], [19]. The lower temperatures of precipitation incubation used in other studies [15], [16], [28] might increase viscosity of the solution lowering the efficiency of centrifugation [19].

As an alternative to ethanol, one volume of 100% isopropanol is often used to precipitate DNA because the precipitation efficiency of this chemical is higher than that of ethanol [13], [29]. Our test with isopropanol to precipitate DNA from the supernatant of CTAB buffer showed the same trends of effects of isopropanol volume, temperature, and incubation time on the yield rate (HC, unpublished data). Isopropanol is less volatile than ethanol and, therefore, requires more time to air-dry samples in the final step. The pellet might also be less-tightly attached to the bottom of the tube when isopropanol is used [29]. Although the incubation time was not a significant factor on the efficiency of precipitation in this study, overnight incubation is recommended for very short length and small amounts of DNA (<15 µg) [20]. In such cases, use of carriers like tRNA, glycogen, or linear polyacrylamide can greatly improve recovery [6], [17].

Yield and quality of extracted DNA depend greatly on the quality of the starting materials. The five methods compared in this study are suitable to extract DNA from a variety of preserved specimens including alcohol-preserved samples or air-dried museum speciments. We used the SDS method to extract DNA from 25-year-old pinned western corn rootworm beetles by immersion or microinjection [18], [30], [31]. The DNA yields from preserved specimens ranged from 3–5 µg /beetle, although the DNA appeared as smears (100–200 bp in size) visualized on an agarose gel suggesting significant degradation. To better prepare dry samples for DNA extraction, fresh insects should first be fixed in 70–100% ethanol for 10–30 min and then be air-dried. A certain degree of DNA degradation is common with unfrozen insect samples, but degraded DNA can be removed by 0.5% agarose gel electrophoresis and re-extraction from the gel.

## Materials and Methods

### Sample collection and preparation

Beetles were collected from the Experimental Farm of the University of Nebraska Haskell Agricultural Laboratory in Concord, Nebraska, USA, in August, 2009. Provided only with water, the beetles were maintained for 2 d under ambient conditions. Individuals were sexed, weighed, and frozen in separate 1.5 ml microfuge tubes at −80°C before DNA extraction.

### DNA extraction protocols

For each method, total DNA was individually extracted from 10 beetles, including 5 females and 5 males. The color of the DNA pellet in each tube was recorded. The DNA from single beetles was re-suspended in 100 µl of molecular-grade water except for the DNA obtained using the DNeasy® Kit which comes with its elution buffer. The DNA solutions were stored at −20°C until further analysis.

The SDS buffer consisted of 0.5% (w/v) SDS diluted in 200 mM Tris, 25 mM EDTA and 250 mM NaCl [1]. In a 1.5 ml microfuge tube with 150 µl of SDS buffer, the beetles were individually grounded using a pestle driven by a handheld electric mixer, then 350 µl of SDS buffer and 5 µl of RNase A solution (100 mg/ml) were added. After incubation at 37°C for 1 hr, 5 µl of Proteinase K solution (20 mg/ml) was added with additional incubation at 50°C for 1 hr. The homogenate was then extracted with 240 µl of phenol/chloroform/isoamyl alcohol (25∶24∶1) and was centrifuged at 12,000 × g for 10 min. The supernatant was transferred into a new 1.5 ml clear-colored tube. To precipitate DNA, 500 µl chilled absolute ethanol was added, and the tube was centrifuged at 12,000 g for 15 min. The pellet was washed twice with 500 µl of 70% ethanol, and centrifuged at the above condition for 3 min to remove residual salt. The pellet was dried in an Eppendorf Vacfuge™ (Eppendorf North America, Hauppauge, NY, USA) at 37°C for 30 min or air-dried at room temperature overnight.

The CTAB buffer consisted of 2% (w/v) CTAB diluted in 100 mM Tris-HCl, 20 mM EDTA, and 1.4 M NaCl; 0.2% (v/v) β–mercaptoethanol was added immediately before use [1]. The tissue lysis, Proteinase K and RNase A treatments, DNA isolation, precipitation, wash and hydration steps were performed as described for the SDS method.

Based on the use of a guanidine-detergent lysing solution that hydrolyzes RNA and allows the selective precipitation of DNA from a cell lysate, DNAzol® (Molecular Research Center, Inc., Cincinnati, OH, USA) is a ready-to-use reagent. Individual beetles were homogenized in 500 µl of DNAzol® reagent with 5 µl of Proteinase K solution (20 mg/ml). Lysis was performed at room temperature for 2 hrs following the manufacturer's protocol [17]. DNA precipitation and drying procedures were the same as in the SDS method.

The Puregene® kit contains two main reagents: cell lysis and protein precipitation solutions (Gentra Systems, Minneapolis, MN, USA). For each beetle, 500 µl of the lysis solution and 5 µl of Proteinase K solution (20 mg/ml) were used. After homogenization, the lysate was incubated at 65°C for 20 min. The procedures of cell lysis, RNase A treatment, and protein precipitation followed the manufacturer's protocol for *Drosophila melanogaster* with necessary modifications according to the beetle weight range [6]. DNA precipitation and drying were done as in the SDS method.

The DNeasy® Mini Kit comprised the mini-spin columns, lysis buffer, two wash buffers, and an elution buffer (Qiagen, Hilden, Germany). Each beetle was lysed using 180 µl Buffer ATL, grounded and incubated at 56°C for 1 hr. Proteinase K and RNase A were added, following the manufacturer's spin-column protocol for animal tissues [7]. To maximize DNA yield, two successive elution steps, each with 50 µl elution buffer, were performed.

### DNA quality and quantity

A NanoDrop® ND1000 Spectrophotometer (NanoDrop Technologies, Inc., Wilmington, DE, USA) was used to measure the DNA concentration and the absorbance ratio (A260/A280). When DNA is extracted from biological samples, protein frequently remains in the DNA solution. Protein is tightly bound to DNA, and complete removal of protein is not always possible. In general, the peak of UV absorption is at 260 nm for DNA and at 280 nm for protein. Thus, when a solution contains both DNA and protein, absorbance at 260 nm is mainly due to the DNA present, and absorbance at 280 nm is due to protein. A pure sample of DNA has the ratio at 1.8 and is relatively free from protein contamination. Generally, the expected ratios for extracted DNA samples should range from 1.7–2.0 [15], [21].

To compare the efficiency of the DNA extraction methods, the DNA yield from single beetles was calculated based on the DNA concentration and final volume. Due to the various body weights of beetles used in different methods, the DNA yield from single beetles was converted into a DNA yield rate (DNA ng/body weight mg). It was assumed that the beetles of the same gender at the identical physical status contained a consistent DNA concentration (ng/mg). Therefore, the yield rates of individual beetles could statistically be compared between female and male and across the methods.

To visualize DNA quality, 250 ng of each DNA was loaded on a 0.5% agarose gel at 45 volts for 2 hrs. The extracted DNA sizes were estimated using the DNA marker of GeneRuler™1 kb Plus (Fermentas, Glen Burnie, MD, USA). A digital image was taken under UV light in a Universal Hood II (Bio-Rad, Hercules, CA, USA).

### PCR amplification of microsatellite loci

To assess the DNA quality for PCR application, a core set of six microsatellite markers (Dvv-D2, Dvv-D4, Dvv-D8, Dvv-T2, Dba05 and Dba07) were amplified from each DNA samples following the protocol of Kim et al. [32].

### Estimation of cost and time required by each method

The cost for each method was estimated based on the price of chemicals, enzymes, and disposable items (including microfuge tubes and pipette tips) consumed for one extraction from a single beetle. The cost ranges were generated by different prices for various package sizes of the above supplies.

The time required to finish one extraction from a single beetle using each method was estimated based on the procedures used in this study, including the time for lysis and 30 min for DNA drying if necessary. The time spent for solution preparation in the SDS and CTAB methods was excluded.

### Optimization of DNA precipitation

A separate group of beetles including 3 females and 3 males was used for individual total DNA extraction using the SDS method. In order to collect enough volume of supernatant containing DNA for the treatments, 600 µl SDS solution was added for each individual extraction. The supernatant from one single beetle was aliquoted into 15 1.5 ml tubes, each with 30 µl of the supernatant. We assumed that the supernatants in the 15 tubes contained an equal amount of DNA. The tubes were treated under a variety of conditions as shown in Table 2.

**Table 2:** Treatments of ethanol volume, incubation temperature and time on DNA precipitation.

	60 µl	120 µl	240 µl
4°C	0 hr	0 hr	0 hr
−20°C	1 hr	1 hr	1 hr
−20°C	15 hr	15 hr	15 hr
−80°C	1 hr	1 hr	1 hr
−80°C	15 hr	15 hr	15 hr

To evaluate the effect of ethanol volume on DNA precipitation from the lysate, three different ratios of 100% ethanol to lysate volume (1∶2, 1∶4 and 1∶8) were tested. After adding ethanol, the tubes were inverted 10 times before the next step and either centrifuged or incubated in −20°C or −80°C. After centrifugation at 12,000 *g* for 15 min, the DNA pellet was washed and dried as described in the SDS method. The DNA pellet in each tube was re-suspended in 30 µl molecular-grade water for further analysis.

### Data analyses

Linear regression and its relevant statistics were used to test the effect of body weight on DNA yield for each extraction method. The general linear model (GLM) was applied to test the effects of extraction method and gender on the DNA yield rate and on the absorbance ratio, respectively. Tukey's pairwise comparisons with the confidence interval of 95% were used to compare the rates or the ratios between the methods. GLM was also applied to test precipitation conditions on DNA yield and main effects plot was generated for ethanol volume and temperature evaluated in this study. The statistical analyses were accomplished using the MINITAB® software Release 14.20 [33].
